# Neighborhood Deprivation and Risk of Congenital Heart Defects, Neural Tube Defects and Orofacial Clefts: A Systematic Review and Meta-Analysis

**DOI:** 10.1371/journal.pone.0159039

**Published:** 2016-10-26

**Authors:** Séverine Deguen, Wahida Kihal, Maxime Jeanjean, Cindy Padilla, Denis Zmirou-Navier

**Affiliations:** 1 EHESP School of Public Health, Department of Environmental and Occupational Health, Rennes, Cedex 35043, France; 2 INSERM U1085 (IRSET), Department of Environmental and Occupational Health, Rennes, Cedex 35043, France; 3 Lorraine University Medical School, Nancy, Cedex 54052, France; Medical University of South Carolina, UNITED STATES

## Abstract

**Background:**

We conducted this systematic review and meta-analysis to address the open question of a possible association between the socioeconomic level of the neighborhoods in which pregnant women live and the risk of Congenital Heart Defects (CHDs), Neural Tube Defects (NTDs) and OroFacial Clefts (OFCs).

**Methods:**

We searched MEDLINE from its inception to December 20^th^, 2015 for case-control, cohort and ecological studies assessing the association between neighborhood socioeconomic level and the risk of CHDs, NTDs and the specific phenotypes Cleft Lip with or without Cleft Palate (CLP) and Cleft Palate (CP). Study-specific risk estimates were pooled according to random-effect and fixed-effect models.

**Results:**

Out of 245 references, a total of seven case-control studies, two cohort studies and two ecological studies were assessed in the systematic review; all studies were enrolled in the meta-analysis with the exception of the two cohort studies. No significant association has been revealed between CHDs or NTDs and neighborhood deprivation index. For CLP phenotype subgroups, we found a significantly higher rate in deprived neighborhoods (Odds Ratios (OR) = 1.22, 95% CI: 1.10, 1.36) whereas this was not significant for CP phenotype subgroups (OR = 1.20, 95%CI: 0.89, 1.61).

**Conclusion:**

In spite of the small number of epidemiological studies included in the present literature review, our findings suggest that neighborhood socioeconomic level where mothers live is associated only with an increased risk of CLP phenotype subgroups. This finding has methodological limitations that impede the formulation of firm conclusions, and further investigations should confirm this association.

## Introduction

Congenital anomalies are a recognized risk factor for stillbirth and neonatal mortality[1]. The contribution made by congenital anomalies to deaths among children under five years of age was estimated at about 10% [1]. Between 2006 and 2010, the European Surveillance of Congenital Anomalies network [2] reported a perinatal mortality rate of 0.81 per 1,000 births associated with congenital anomalies (of which 27% were due to chromosomal anomalies, 27% to perinatal deaths, 24% to CHDs and 16% to anomalies of the nervous system). The determinants of half of all major malformations are multifactorial, featuring environmental nuisance factors (air pollution, proximity to landfills)[3–6], socioeconomic factors [7], and poor access to amenities such as health care services during pregnancy[8].

Congenital malformations are giving rise to growing public and scientific concern. The impact of maternal characteristics on specific congenital abnormalities is well documented [9–13]. A recent meta-analysis[7] reported that maternal educational attainment and occupation as well as household income were associated with an increased risk of CHDs. However, the majority of these studies considered deprivation measures at an individual level, without considering the neighborhoods in which people lived.

Yet a number of studies have noted that beyond individual characteristics, the place or neighborhood in which people live may influence the way in which health operates through such mechanisms as: (i) availability and accessibility of amenities (including health care services, leisure and recreational facilities such as parks & green spaces, stores selling healthy and non-healthy food), (ii) life stress and (iii) social support or social cohesion[14].

Several studies have suggested an association between increased risks of adverse reproductive outcomes and socioeconomic neighborhood level [15–17], revealing for instance that infant mortality risk is higher among women living in the most deprived neighborhoods, in comparison with those living in the most privileged areas (Relative Risks (RR) = 2.62, 95% CI: 1.87, 3.70)[18], or that women living in the lowest neighborhood income quintile are significantly more likely to have a preterm birth than those living in the wealthiest neighborhood income quintile (OR = 1.14, 95% CI: 1.10, 1.17)[16].

In this context, further exploration of the risk of congenital anomalies in relation to neighborhood deprivation seems relevant. The aim of this systematic review, followed by a meta-analysis, is to assess whether the current epidemiological evidence is in favor of an association between congenital malformations and living in deprived neighborhoods, with a view to suggesting future directions for research.

## Methods

### Search strategy

A systematic literature search was conducted using the PubMed platform providing access to the MEDLINE and Academic Search Complete databases, among articles published up until December 20^th^ 2015. The search strategy followed the PRISMA (Preferred Reporting Items for Systematic reviews and Meta-Analyses) guidelines [19] and was performed with the following keywords found in article titles:

Heart Defects [Title/Abstract] or Heart Defect [Title/Abstract] or Heart Diseases [Title/Abstract] or Heart Disease [Title/Abstract] or cleft [Title/Abstract] or clefts [Title/Abstract] or neural tube defects [Title/Abstract] or neural tube defect [Title/Abstract]) and (birth or births or pregnancy or congenital [Title/Abstract]) and (deprivation [Title] or socio-economic [Title] or socioeconomic [Title] or socioeconomics [Title] or inequality [Title] or inequalities [Title] or contextual [Title] or disadvantage [Title] or disadvantages [Title] or disadvantaged [Title] or advantage [Title] or advantages [Title] or advantaged [Title] or income [Title] or employment [Title] or unemployment [Title] or neighbourhood [Title] or neighborhood [Title] or rural [Title] or urban [Title] or rural and urban [Title] or lifestyle [Title] or socio-occupational [Title] or insurance [Title] or educational [Title] or social [Title] or healthcare [Title]).

### Selection of studies

**Fig 1:**
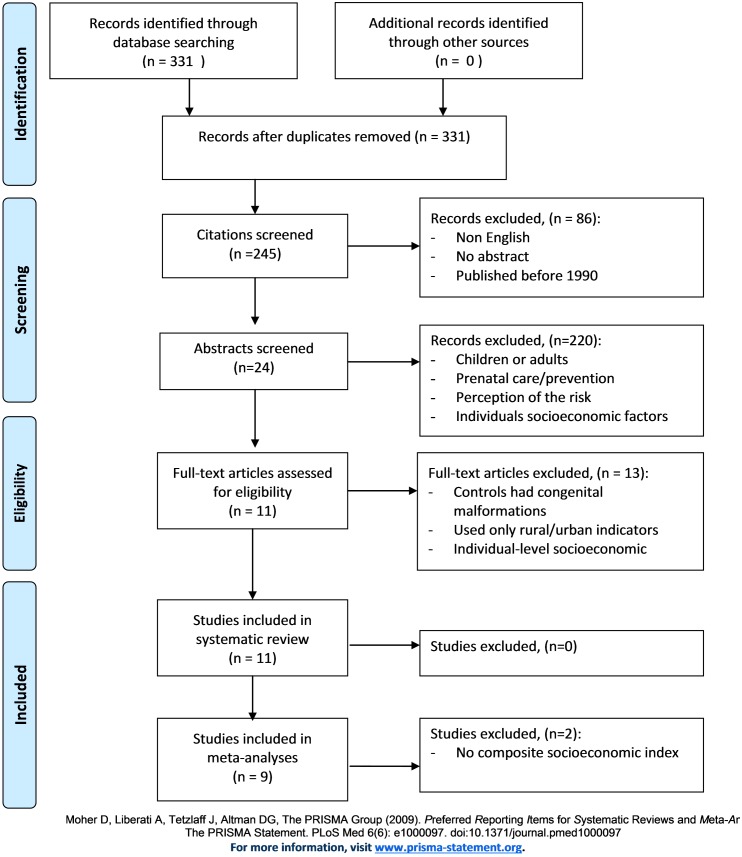
Flow diagram for inclusion and exclusion of studies. Caption Details the different steps of the selection process, in line with PRISMA recommendations.

Fig 1 summarise the different steps of the selection process, in line with PRISMA recommendations. At the first step, the inclusion criteria were peer-reviewed papers written in English and articles published after 1990 without restriction on geographical location. We restricted our systematic review to the three main groups of congenital abnormalities, namely: CHDs, NTDs and OroFacial Clefts (OFCs). Papers presenting non-original studies (e.g. comments, case reports, animal and mechanistic studies and biological experiments) were ultimately excluded. In all, 245 of the 331 articles published were selected.

At the second step, abstracts of the 245 studies were screened manually by two independent experts (SD and WK, authors of this article); studies were excluded when they:

- were performed on an adult population rather than on newborns or infants
- investigated death or hospitalization among the population having congenital malformations
- dealt with health system care and particularly the impact made by medical care during pregnancy on the risk of congenital malformation
- were interested in the perception of risk related to congenital malformations
- considered socioeconomic factors measured at individual level only

Full manuscripts of the remaining 24 articles (of the 245 initially selected) were thoroughly checked. 13 were then excluded because:

- the rural/urban indicators used could not be related to socioeconomic level
- the control group was defined as babies with a congenital malformation
- the studies used individual socioeconomic indicators that had not been detected after reading only the abstract

Ultimately, a total of 11 articles met the inclusion criteria for the systematic literature review.

Lastly, in order to reduce heterogeneity between studies, we decided to include studies that used a composite metric to measure socioeconomic deprivation in the meta-analysis. Hence, of the 11 articles included in this systematic literature review, two did not meet the inclusion criteria for the meta-analysis. In the end, nine articles were included in the meta-analysis.

### Extraction data

For each study, the following information was extracted and reported in Tables 1–3: General Information (first author's name, date of study and country of origin), Main Study Characteristics (study design, spatial unit, statistical methods, population definition, database, main findings), Participant Characteristics (information on confounders, neighborhood deprivation measures), and Outcome Measures (outcomes classification and definition). Wherever possible, we reported measures from the adjusted models presented in each study. OR and similar metrics measuring the strength of association between congenital malformation and neighborhood socioeconomic deprivation were also extracted. In the absence of indicators measuring the strength of association between congenital malformations and neighborhood socioeconomic deprivation, we estimated the odds ratio or equivalent where information was available in the article. For cohort studies, we considered risk ratios to give equivalent results to OR, given the rarity of the outcome.

**Table 1:** Main characteristics of the selected studies

Source	Design, period, location	Congenital malformation^a^	Spatial unit^b^	Neighborhood deprivation measure^c^	Confounders/ Matching factors	Statistical methods	Main findings
**Lupo PJ, 2015**[22]	CC, 1999–2008, Texas	***Specific defects*,** CLP and CP	Census tract	Six socio-economic variables: Poverty, education, unemployment, service or production occupation, rental occupancy, crowding, neighborhood deprivation index (NDI)	Matched by year of birth; Babies characteristics: sex and birth of year—Mothers characteristics: age, race/ethnicity, education—Mothers behavior: smoking	Mixed effects; logistic regression	Deprived socioeconomic positions were significantly associated with an increased risk of CLP except for crowding—No significant association has been revealed with CP.
**Pawluk MS, 2014**[23]	CC, 1992–2001, Argentina	***Specific defects*** SB and An, CLP and CP, TA and VSD	Region	Regional Socio-economic level based on the Unmet Basic Need (UBN) index	Matched by time and place of birth—Mothers characteristics: age, gravidity order, native descent—Mothers behavior: number of antenatal visits	Multilevel logistic regression	CLP was significantly associated with a lower socioeconomic level—No significant association has been revealed with others specific defects.
**Carmichael SL, 2009**[9]	CC, 1999–2004, USA	***Specific defects;*** CLP and CP dTGA and TOF	Census tract, block group	Six socio-economic variables: Poverty, education, unemployment, service or production occupation, rental occupancy, crowding, socioeconomic index	Mothers characteristics: race-ethnicity, body mass index—Mothers behavior: intake of folic acid-containing supplements, smoking, binge drinking	Logistic regression	dTGA and TOF were not significantly associated with any socioeconomic variable—Results of CP suggested that worse socioeconomic level was associated with decreased risk whereas for CLP, all ORs were not statistically significant.
**Grewal J, 2009**[24]	CC, 1999–2003, USA	NTDs overall, ***Specific defects*** SB and An	Census tract, Block group	Six socio-economic variables: Poverty, education, unemployment, service or production occupation, rental occupancy, crowding, socioeconomic index	Matched on birth hospital—Mothers characteristics: Age, body mass index, gravidity, race-ethnicity—Mothers behavior Intake of folic acid-containing supplements	Logistic regression	NTDs overall and subtypes were not significantly associated with any socioeconomic variable.
**Durning P, 2007**[25]	Ecological, 1982–2003, Wales	OFCs overall, ***Specific defects*** CLP and CP	Ward	Townsend index	None	Wilson’s method; Chi-square tests	The risk of OFC, CLP and CP increased significantly with the increase of deprivation level.
**Clark JD, 2003**[26]	Ecological, 1989–1998, Scotland	OFCs overall, ***Specific defects*** CLP and CP	Postcode sector	Carstairs index	None	Chi-square tests	OFC, CLP and CP were all significantly associated with a lower socioeconomic level.
**Carmichael SL, 2003**[27]	CC, 1987–1989, USA	***Specific defects*** CLP and CP, dTGA and TOF	Census tract, Block group	Six socio-economic variables: Poverty, education, unemployment, service or production occupation, rental occupancy, crowding, socioeconomic index	Mothers characteristics: Race-ethnicity—Mothers behavior—Vitamin use, smoking, binge drinking	Logistic regression	No significant association has been revealed with CP, CLP and dTGA whatever the socioeconomic indicator—the risk of TOF decreases significantly with the percentage of unemployment and with the level of corwding.
**Vrijheid M, 2000**[28]	CC, 1986–1993, UK	NTDs overall, CHDs overall, OFCs overall ***Specific defects*** Chambers and connections, Cardiac septa, Cardiac valves of great arteries and vein, CLP and CP	Enumeration district	Carstairs index	Matched on year of birth, study area—Neighborhood characteristics: distance of residence from a landfill—Mother’s characteristics: age	Logistic regression	Results suggest that deprived neighborhoods have higher rates of cardiac septa—No significant result has been revealed for others type of malformations.
**Wasserman CR, 1998**[11]	CC, 1989–1991, USA	NTDs overall	Census tract, Block group	Six socio-economic variables: Poverty, education, unemployment, service or production occupation, rental occupancy, crowding, socioeconomic index	Matched on the residence country of the mother—Mother’s characteristics: race/ethnicity, age, BMI, fever, education, household income, employment, family occupation—Mothers behavior: periconceptional vitamin use	Logistic regression	NTDs were significantly associated with a lower socioeconomic level.
**Agha MM, 2013**[13]	Co, 1994–2009, Canada	NTDs overall	Enumeration area, Dissemination area	two socio-economic variables: income, education level	Babies characteristic: sex—Mothers characteristics: Age—Others: time era	logistic regression	NTDs were significantly associated with both low education level and low income
**Agha MM, 2011**[29]	Co, 1994–2007, Canada	CHDs overall, CHDs severe and CHDs non severe	Enumeration area, Dissemination area	two socio-economic variables: income, education level	Babies characteristic: sex—Mothers characteristics: Age, history of diabetes—Others: time era, very low birth weight	logistic regression	CHDs were significantly associated with both low education level and low income—Non-severe CHDs were also significantly associated with education and income while severe CHDs not.

^a^ Congenital malformations: CHDs (Congenital Heart Defects); TA (Truncus Arteriosus); VSD (Ventricular Septal Defect); dTGA (dextro-Transposition of the Great Arteries); TOF (Tetralogy Of Fallot); OFCs (OroFacial clefts); CP (Cleft palate); CLP (Cleft Lip with or without cleft Palate); NTDs (Neural tube Defects); An (Anencephaly); SB (Spina Bifida).

^b^ Spatial unit: Small-Area Market Statistics (SAMS) (contain an average of approximatively 1000 residents); Enumeration district (approximately 150 households); Enumeration and dissemination areas (average of 282 households); census block group and census tract (varying approximately between 5000 and 1000 households); region, ward and postcode sector (no information about size or number of inhabitants/households).

^c^ See definition of neighborhood deprivation measure in Table 3.

**Table 2:** Definitions of congenital malformation outcomes and studied population.

	Type(s) or subtype(s) of malformation(s)	Outcome classification^a^	Population study	Database source of congenital malformations	Authors, year
**Congenital Heart Defects (CHDs)**	Overall heart defects	ICD 9; ICD 10	All children born alive in a hospital	Discharge Abstract Database of the Canadian Institute for Health Information (CIHI-DAD)	Agha MM, 2011
		ICD 9; ICD 10; EUROCAT subgroups definition	Cases: live births, stillbirths, fetal deaths from 20 weeks gestation, terminations of pregnancy—Controls: non-malformed live births	**T**hree regional UK registers: Glasgow, Northern Region and North Thames West	Vrijheid M, 2000
	Severe heart defects	ICD 9; ICD 10	All children born alive in a hospital	Discharge Abstract Database of the Canadian Institute for Health Information (CIHI-DAD)	Agha MM, 2011
	Non severe heart defects	ICD 9; ICD 10	All children born alive in a hospital	Discharge Abstract Database of the Canadian Institute for Health Information (CIHI-DAD)	Agha MM, 2011
	Ventricular septal defects	NC	Cases: newborn, stillbirths (weight>500grams)—Controls: non-malformed live births	Latin-American Collaborative Study of Congenital Malformations (ECLAMC)	Pawluk MS, 2014
	Truncus arteriosus				
	Cardiac septa	ICD 9; ICD 10; EUROCAT subgroups definition	Cases: live births, stillbirths, fetal deaths from 20 weeks gestation, terminations of pregnancy—Controls: non-malformed live births	**T**hree regional UK registers: Glasgow, Northern Region and North Thames West	Vrijheid M, 2000
	Cardiac valves				
	Great arteries and veins				
	Chambers and connections				
	Dextro-transposition of the great arteries	British Pediatric Association (BPA) coding system based on ICD 9	Cases: Live births, stillbirths (fetal deaths from 20 weeks gestation), prenatally diagnosed terminations of pregnancy—Controls: non-malformed live births	Multiple hospital reports and medical records	Carmichael SL, 2009
		Pathogenic classification scheme of Clark	Cases: infants and fetal deaths—Controls: Live born infants	California Birth Defects Monitoring Program (CBDMP)	Carmichael SL, 2003
	Tetralogy of Fallot	British Pediatric Association (BPA) coding system based on ICD 9	Cases: Live births, stillbirths (fetal deaths from 20 weeks gestation), prenatally diagnosed terminations of pregnancy—Controls: non-malformed live births	Multiple hospital reports and medical records	Carmichael SL, 2009
		Pathogenic classification scheme of Clark	Cases: infants and fetal deaths—Controls: Live born infants	California Birth Defects Monitoring Program (CBDMP)	Carmichael SL, 2003
**Neural Tube Defects (NTDs)**	Overall NTDs	ICD 9; ICD 10	All children born alive in a hospital	Discharge Abstract Database of the Canadian Institute for Health Information (CIHI-DAD)	Agha MM, 2013
		NC	Cases: Live births, stillbirths (fetal deaths from 20 weeks gestation), prenatally diagnosed terminations of pregnancy—Controls: non-malformed live births	Multiple hospital reports and medical records	Grewal J, 2009
		ICD 9; ICD 10; EUROCAT subgroups definition	Cases: live births, stillbirths, fetal deaths from 20 weeks gestation, terminations of pregnancy—Controls: non-malformed live births	**T**hree regional UK registers: Glasgow, Northern Region and North Thames West	Vrijheid M, 2000
		ICD-9	Cases: live births, fetal deaths—Controls: non-malformed singleton infants	California Birth Defects Monitoring Program (CBDMP)	Wasserman CR, 1998
	Spina bifida and Anencephaly	NC	Cases: newborn, stillbirths (weight>500grams)—Controls: non-malformed live births	Latin-American Collaborative Study of Congenital Malformations (ECLAMC)	Pawluk MS, 2014
		NC	Cases: Live births, stillbirths (fetal deaths from 20 weeks gestation), prenatally diagnosed terminations of pregnancy—Controls: non-malformed live births	- Multiple hospital reports and medical records	Grewal J, 2009
**OroFacial Clefts (OFC)**	Overall OFCs	NC	Live births	South, West, and Central Wales Orofacial-Cleft Register	Durning P, 2007
		NC	Live births	**R**egister of the Cleft Service in Scotland (CLEFTSiS)	Clark JD, 2003
		ICD 9; ICD 10; EUROCAT subgroups definition	Cases: live births, stillbirths, fetal deaths from 20 weeks gestation, terminations of pregnancy—Controls: non-malformed live births	**T**hree regional UK registers Glasgow, Northern Region and North Thames West	Vrijheid M, 2000
	Cleft lip with/without palate and Cleft Palate	National Birth Defects PreventionStudy	Cases: live births, spontaneous fetal deaths, pregnancy terminations—Controls: non-malformed live births	Texas Birth Defects Registry	Lupo PJ, 2015
		NC	Cases: newborn, stillbirths (weight>500grams)—Controls: non-malformed live births	Latin-American Collaborative Study of Congenital Malformations (ECLAMC)	Pawluk MS, 2014
		British Pediatric Association (BPA) coding system based on ICD 9	Cases: Live births, stillbirths (fetal deaths from 20 weeks gestation), prenatally diagnosed terminations of pregnancy—Controls: non-malformed live births	Multiple hospital reports and medical records	Carmichael SL, 2009
		Pathogenic classification scheme of Clark	Cases: infants and fetal deaths—Controls: Live born infants	California Birth Defects Monitoring Program (CBDMP)	Carmichael SL, 2003
		NC	Live births	**R**egister of the Cleft Service in Scotland (CLEFTSiS)	Clark JD, 2003
		ICD 9; ICD 10; EUROCAT subgroups definition	Cases: live births, stillbirths, fetal deaths from 20 weeks gestation, terminations of pregnancy—Controls: non-malformed live births	Three regional UK registers Glasgow, Northern Region and North Thames West	Vrijheid M, 2000

^a^Outcome Classification: NC = no classification or no stated; ICD = International classification of disease (version 9 and 10); Pathogenic classification scheme of Clark [30]

**Table 3:** Definition of neighborhood deprivation measures—The definitions were entirely copied out from the original articles.

Authors, year	Poverty	Education	unemployment	Service/production occupation	Rental occupancy	Crowding	Socioeconomic index
Lupo PJ, 2015	Proportion of households below the poverty level in 1999	Proportion of the population without a high school diploma or equivalent	Proportion of the population unemployed in 1999	Proportion of employed civilian population aged at least 16 years that is employed in a service or production occupation	Proportion of occupied housing units that are occupied	Number of occupants per room in a household	This index includes poverty, education, unemployment, service or production occupation, rental occupancy and crowding
Grewal J, 2009	Proportion of non- institutionalized population living below the poverty level, which was $17029 for a family of 4 in 1999	Proportion of the population aged ≥ 25 without a high school diploma or equivalent	Proportion of the population aged ≥ 16 that is not working	Proportion of employed population aged ≥ 16 in occupations that include operators, fabricators and laborers	Proportion of occupied housing units that are rented	Proportion of occupied housing units with an average of more than one person per room	This index includes poverty, education, unemployment, service or production occupation, rental occupancy and crowding
Carmichael SL, 2009	Proportion of non- institutionalized population living below the poverty level, which was $17029 for a family of 4 in 1999	Proportion of the population aged ≥ 25 without a high school diploma or equivalent	Proportion of the population aged ≥ 16 that is not working	Proportion of employed population aged ≥ 16 in occupations that include operators, fabricators and laborers	Proportion of occupied housing units that are rented	Proportion of occupied housing units with an average of more than one person per room	This index includes poverty, education, unemployment, service or production occupation, rental occupancy and crowding
Carmichael SL, 2003	Proportion of non- institutionalized population living below the poverty level, which was $12674 for a family of 4 in 1989	Proportion of the population aged ≥ 18 without a high school diploma or equivalent	Proportion of the population aged ≥ 16 not employed	Proportion of employed population aged ≥ 16 in occupations that include operators, fabricators and laborers	Proportion of occupied housing units that are renter occupied	Proportion of occupied housing units with an average of more than one person per room	This index includes poverty, education, unemployment, service or production occupation, rental occupancy and crowding
Wasserman CR, 1998	Proportion of non- institutionalized population living below the federal poverty level	Proportion of the population aged ≥ 18 who did not graduate from high school	Proportion of the population aged ≥ 16 not employed	Proportion of employed population aged ≥ 16 in occupations that include operators, fabricators and laborers	Proportion of all occupied housing units that are renter occupied	Proportion of all occupied housing units with greater than 1 person per room	This index includes poverty, education, unemployment, service or production occupation, rental occupancy and crowding
Pawluk MS, 2014							A regional socioeconomic index based on the unmet basic need (UBN) index
Durning P, 2007							The Townsend index includes percentage of unemployed, percentage of households without a car, percentage of households not owner occupied and percentage of households overcrowded.
Clark JD, 2003							The Carstairs index includes the four following variables: (A) Overcrowding: persons in private households living at a density of > 1 person per room as a proportion of all persons in private households (B) Male unemployment: proportion of economically active males who are seeking work (C)Low social class: proportion of all persons in private households with head of household in social class 4or 5 (D) No car: Proportion of all persons in private households with no car
Vrijheid M, 2000							Carstairs index (no more details given in the article)

### Meta-analysis

Associations were examined for NTDs, CHDs and OFCs overall, as well as for any of their subtypes. For studies reporting risk estimates in association with neighborhood socioeconomic deprivation for more than one outcome, each meta-risk estimate was computed on its own, using the specific socioeconomic status (SES) metric.

The meta-association between a neighborhood socioeconomic deprivation measure and congenital anomalies was computed only where at least four studies were available. RR and OR, according to the study design, were used as the summary association measure between the metric of neighborhood socioeconomic deprivation and CHDs, OFCs or NTDs. Because socioeconomic metrics were always reported as categorical metrics, combined ORs were computed as contrasts between most- versus least- deprived quantiles.

To obtain the combined effect, both fixed and random effects models can be used. Different assumptions about the nature of the studies are formulated and these lead to different definitions of the combined effect, and different formulas for assigning weights. Using the fixed-effect model, we will assume that there is one true effect size estimated from all included studies, whereas using the random-effect model we allow that the true effect could vary from one study to the next. To determine which model is most appropriate, we assessed the heterogeneity degree of associations between neighborhood socioeconomic deprivation metrics and congenital anomaly outcomes, using the Cochran Q-test to find out whether heterogeneity is statistically significant. When the p-value of the Q-test is lower than 0.1, the random (rather than fixed) effect model was used to take account of the detected heterogeneity. In addition, heterogeneity between studies was quantified using I^2^ statistic corresponding to the percentage of variation imputable to heterogeneity[20]; an I^2^ value varying between 25% and 50%, 50% and 75% and > 75% corresponding to low, medium and high heterogeneity, respectively[20].

Forest plots were created to draw the combined risk estimates. All statistical analysis was performed using STATA 11.

Funnel plots for the assessment of potential publication bias were not applicable because of the small number of studies. At least ten studies are recommended in the meta-analysis to characterize real asymmetry[21].

## Results

### Main characteristics of the studies

There were 11 studies published from 1998 to 2015, including nine estimating the association between neighborhood socioeconomic deprivation index and congenital malformations and eligible for the meta-analyses. Of these, CHDs and NTDs were investigated in four different studies and OFCs in seven studies (Table 1). In addition, two cohort studies were eligible for the systematic review (1 study of CHDs and one of NTDs, table 1) though not the meta-analyses. About 10, 000 cases were included in the meta-analyses distributed across 1,130 cases of CHDs, 1,499 cases of NTDs and 7,019 cases of OFCs

### Study design and location

Most of the studies were conducted in the United States or Canada (7 studies: [9,11,13,22,24,27,29]). There were also three studies conducted in Europe[25,26,28] and one in Argentina[23]. With the exception of two ecological epidemiology studies[25,26], all analyzed individual data, with seven case-control study design papers[9,11,22–24,27,28] and two cohort studies[13,29] (Table 1).

### Cases and databases

Definition of congenital malformations was heterogeneous across studies (Table 2). A majority of studies based their outcome definition on the International Classification of Disease 9 and 10 (ICD 9–10)[9,11,13,28,29] while others did not [9,11,23–28].

Databases were drawn from specific registers[22,25,26,28], national birth defect prevention studies and monitoring programs [11,13,23,27,29] as well as from hospital reports and medical records[9,24] (Table 2).

While some studies investigated only the overall group of malformations such as NTDs[11,13,24,28,29] others explored specific subtypes: Anencephaly (An) and Spina Bifida (SB)[23,24]. In 2011, Agha *et al*. divided CHDs into severe and non-severe malformations [29], whereas other authors distinguished Ventricular Septal Defects (VSD) and truncus arteriosus [23], as well as malformations of cardiac septa including ASD (Atrial Septal Defect) and VSD, cardiac chambers and connections, cardiac valves, great arteries and veins[28]. In 2003 and 2009, Carmichael *et al*. focused on both conotruncal defects: Tetralogy Of Fallot (TOF) and dextro-Transposition of Great Arteries (dTGA)[9,27]. All studies that investigated OFCs studied CLP and CP, separately [9,22,23,25–28]. On the whole, CLP and CP subtypes seem to be the most investigated defect phenotype subtypes, followed by An and SB defects.

### Neighborhood level

American studies used census tract and block level [9,11,22,24,27] as well as enumeration or dissemination area[13,29] whereas UK studies had information at the level of enumeration districts [28], postcode sector [26], or ward [25]. One study has recently used a wider level—the region—to explore the association between deprivation and specific defects such as SB, anencephaly, CP, CLP, ventricular septal defect and truncus arterious[23].

### Deprivation assessment

Various indicators were used to assess neighborhood deprivation (Table 3). Most studies used composite indices such as the Townsend score[25], the Carstairs index [26,28], an *ad hoc* regional index [23], or neighborhood indices [9,11,22,24,27]. Studies also used just one socioeconomic variable to characterize contextual deprivation as defined in Table 3: community and household size-adjusted measure of household income [13,29], education level [9,11,22,24,27], poverty [9,11,22,24,27], unemployment[9,11,22,24,27], operator/laborer occupation [9,11,22,24,27], crowding [9,11,22,24,27] and rental occupancy [9,11,22,24,27].

### Statistical analysis

Of the studies relying on individual information, several used conventional logistic regression [9,11,13,24,27–29]. Others investigated the role of neighborhood deprivation beyond individual characteristics by using multilevel logistic regression [22,23]. Clark *et al*., in 2003 and Durning *et al*. in 2007 used only Chi-square tests to assess associations among cleft types and deprivation categories [25,26].

### Confounding factors

A broad set of covariates has been used regarding baby and/or maternal characteristics [9,11,13,22–24,27–29], prenatal and/or postnatal care [23], events of maternal fever/infection [11] or maternal history of diabetes [29] and distance of residence from a landfill site [28]. The most frequently used covariates were maternal age [11,13,22–24,29], body mass index [9,11,24,27] and intake of peri-conceptional supplements containing folic acid [9,11,24,27]. Other studies adjusted on smoking [9,22,27] or binge drinking habits[9,27].

### Main findings

#### Systematic review findings for CHDs

Of the two studies considering all types of CHDs, one shows an increased risk of CHDs for those living in a deprived area, either characterized by low income (OR = 1.05, 95% CI: 1.01, 1.10) or by a low level of education (OR = 1.26, 95% CI: 1.21, 1.32) [29] (table 4). More precisely, in 2011, Agha *et al*. reported that infants born to women living in a neighborhood with low income or a low level of education had a significantly higher risk of non-severe CHDs, (OR = 1.04, 95% CI: 1.0, 1.09) and (OR = 1.28, 95% CI: 1.22, 1.34), respectively [29]. However, this association was not significant in the study by Vrijheid *et al*. (OR = 1.59, 95% CI: 0.98, 2.59) [28].

**Table 4:** Risk associated with CHDs for different neighborhood socioeconomic indicators.

Neighborhood deprivation	Design, date, location	Malformation(s)	OR	95% CI	Authors, Year
Income	Co, 1994–2007, Canada	Non severe CHDS	**1.04**	**[1.00; 1.09]**	**Agha et al. 2011**
		Severe CHDs	1.09	[0.88; 1.36]	
		Overall CHDs	**1.05**	**[1.01; 1.10]**	
Education	Co, 1994–2007, Canada	Non severe CHDS	**1.28**	**[1.22; 1.34]**	**Agha et al. 2011**
		Severe CHDs	1.2	[0.96; 1.50]	
		Overall CHDs	**1.26**	**[1.21; 1.32]**	
	CC, 1999–2004, USA	Dextro-transposition of great arteries	0.7	[0.4; 1.3]	Carmichael et al. 2009
	CC, 1987–1989, USA		2.0	[0.8; 4.8]	Carmichael et al. 2003
	CC, 1999–2004, USA	Tetralogy of fallot	0.7	[0.4; 1.1]	Carmichael et al. 2009
	CC, 1987–1989, USA		0.5	[0.2; 2.1]	Carmichael et al. 2003
Employment	CC, 1999–2004, USA	Dextro-transposition of great arteries	0.7	[0.4; 1.3]	Carmichael et al. 2009
	CC, 1987–1989, USA		1.2	[0.4; 3.0]	Carmichael et al. 2003
	CC, 1999–2004, USA	Tetralogy of fallot	0.9	[0.6; 1.5]	Carmichael et al. 2009
	CC, 1987–1989, USA		**0.3**	**[0.1; 0.8]**	**Carmichael et al. 2003**
Poverty	CC, 1999–2004, USA	Dextro-transposition of great arteries	0.7	[0.4; 1.1]	Carmichael et al. 2009
	CC, 1987–1989, USA		1.5	[0.7; 3.6]	Carmichael et al. 2003
	CC, 1999–2004, USA	Tetralogy of fallot	0.8	[0.5; 1.2]	Carmichael et al. 2009
	CC, 1987–1989, USA		0.4	[0.2; 1.0]	Carmichael et al. 2003
Operator/laborer	CC, 1999–2004, USA	Dextro-transposition of great arteries	0.8	[0.6; 1.6]	Carmichael et al. 2009
	CC, 1987–1989, USA		2.0	[0.9; 4.6]	Carmichael et al. 2003
	CC, 1999–2004, USA	Tetralogy of fallot	0.7	[0.4; 1.1]	Carmichael et al. 2009
	CC, 1987–1989, USA		0.6	[0.3; 1.3]	Carmichael et al. 2003
Crowding	CC, 1999–2004, USA	Dextro-transposition of great arteries	0.8	[0.5; 1.4]	Carmichael et al. 2009
	CC, 1987–1989, USA		1.5	[0.6; 3.4]	Carmichael et al. 2003
	CC, 1999–2004, USA	Tetralogy of fallot	0.7	[0.4; 1.2]	Carmichael et al. 2009
	CC, 1987–1989, USA		**0.4**	**[0.2; 0.9]**	**Carmichael et al. 2003**
Rental occupancy	CC, 1999–2004, USA	Dextro-transposition of great arteries	0.7	[0.4; 1.3]	Carmichael et al. 2009
	CC, 1987–1989, USA		1.4	[0.7; 2.8]	Carmichael et al. 2003
	CC, 1999–2004, USA	Tetralogy of fallot	1.2	[0.8; 1.9]	Carmichael et al. 2009
	CC, 1987–1989, USA		0.6	[0.3; 1.4]	Carmichael et al. 2003
Specific socioeconomic index	CC, 1999–2004, USA	Dextro-transposition of great arteries	0.6	[0.4; 1.1]	Carmichael et al. 2009
	CC, 1987–1989, USA		2.6	[0.9; 7.5]	Carmichael et al. 2003
	CC, 1999–2004, USA	Tetralogy of fallot	0.9	[0.6; 1.5]	Carmichael et al. 2009
	CC, 1987–1989, USA		0.7	[0.2; 2.5]	Carmichael et al. 2003
Carstairs Index	CC, 1986–1993, UK	Overall CHDs	1.59	[0.98; 2.59]	**Vrijheid et al. 2000**
		Cardiac chambers and connections	1.94	[0.53; 7.13]	
		Cardiac septa	**2.82**	**[1.43; 5.56]**	
		Cardiac valve	1.49	[0.66; 3.36]	
		Great veins	1.04	[0.48; 2.23]	
UBN Index	CC, 1992–2001, Argentina	Truncus arteriosus	0.73	[0.44; 1.20]	Pawluk et al. 2014
		Ventricular septal defect	1.06	[0.76; 1.49]	

Among the studies focusing on specific subtypes of CHDs, the results were contrasted both between studies and within a single study. Despite several non-significant associations, few results found on dTGA outcome suggested that women living in deprived neighborhoods were more likely to have children with a dTGA malformation (although the associations were not statistically significant), for instance as assessed by a high deprivation score (OR: 2.6, 95% 0.9, 7.5) or by low education (OR: 2.0, 95% 0.8, 4.8) [27]. In other studies, however, the risk of dTGA tends to fall where women have lived in deprived neighborhoods—for instance as measured by a low level of education (OR: 0.7, 95% CI: 0.4, 1.3) or poverty (OR = 0.7, 95% CI: 0.4, 1.1) [9].

With regard to the TOF malformation, the study by Carmichael *et al*. was the only one to find significant associations. The study revealed a decreasing risk alongside a rise in unemployment percentage (OR: 0.3, 95% 0.1, 0.8) and an increase of overcrowding (OR: 0.4, 95% 0.2, 0.9) [27].

For Vrijheid *et al*. in 2000[28], and Pawluk *et al*., in 2014[23], the risk of specific cardiac malformations tended to be high, though not statistically significant except for cardiac septa subtypes (OR = 2.82, 95% CI: 1.43, 5.56) [28].

#### Meta-analysis for CHDs

**Fig 2:**
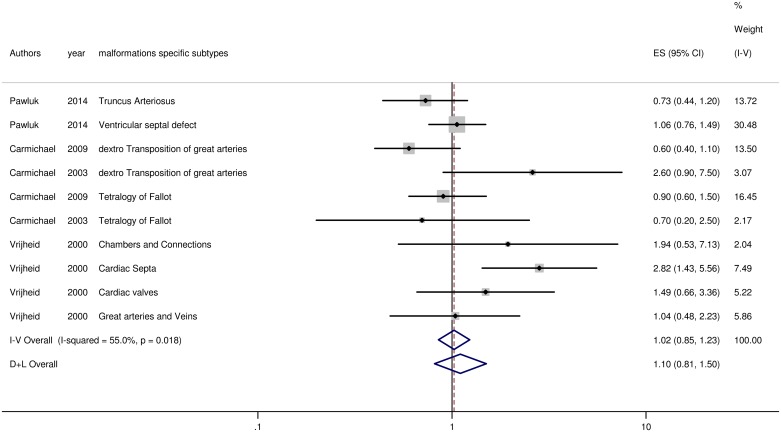
Forest plot on the effect of neighborhood deprivation on CHDs. Caption ES: Effect Size; CI: Confidence Interval

A total of ten results regarding deprivation and cardiac anomalies were included in the meta-analysis (Fig 2). There was significant heterogeneity in the summary-effect estimates for the CHDs group (I^2^ = 55%; p = 0.018) which recommended use of the random-effect model. Results suggest that living in a deprived area is not statistically associated with the risk of CHDs (OR = 1.10, 95% CI: 0.81, 1.50).

#### Systematic review findings for NTDs

Among the four studies considering all NTD types, some results have shown an increased risk for newborns to women living in deprived neighborhoods (OR = 3.2, 95% CI: 1.9, 5.4) [11] measured according to income (OR = 1.2, 95% CI: 1.08, 1.33) [13], education (OR = 1.17, 95% CI: 1.05, 1.3) [13] and (OR = 1.8, 95% CI: 1.1, 2.7) [11], poverty (OR = 1.5, 95% CI: 1.7 CI: 1.0, 2.2), operator/laborer occupation (OR = 1.8, 95% CI: 1.2, 2.7), crowding (OR = 1.7, 95% CI: 1.2, 2.6) or Rental (OR = 1.3, 95% CI: 1.0, 1.8) [11] (Table 5). One study showed inverse (but non-significant) associations for poverty (OR = 0.9, 95% CI: 0.6, 1.4) and rental occupancy (OR = 0.8, 95% CI: 0.5, 1.2)[24]. Regarding NTD subtypes, all results are non-significant and contrasted both between studies and within a single study. No specific trend was observed for SB and An defects.

**Table 5:** Risk associated with NTDs for different neighborhood socioeconomic indicators.

Neighborhood deprivation	Design, date, location	Malformation(s)	OR	95% CI	Authors, Year
Income	Co, 1994–2009, Canada	Overall NTDs	**1.20**	**[1.08; 1.33]**	**Agha et al. 2013**
Education	Co, 1994–2009, Canada	Overall NTDs	**1.17**	**[1.05; 1.3]**	**Agha et al. 2013**
	CC, 1989–1991, USA		**1.8**	**[1.1; 2.7]**	**Wasserman et al 1998**
	CC, 1999–2003, USA		1.0	[0.7; 1.6]	Grewal et al. 2009
	CC, 1999–2003, USA	Spina bifida	0.9	[0.5; 1.5]	Grewal et al. 2009
		Anencephaly	1.2	[0.7; 2.1]	
Employment	CC, 1999–2003, USA	Spina bifida	1.2	[0.7; 1.9]	Grewal et al. 2009
		Anencephaly	1.2	[0.7; 2.1]	
	CC, 1999–2003, USA	Overall NTDs	1.2	[0.8; 1.8]	Grewal et al. 2009
	CC, 1989–1991, USA		1.3	[0.9; 1.8]	Wasserman et al. 1998
Poverty	CC, 1999–2003, USA	Spina bifida	0.8	[0.5; 1.4]	Grewal et al. 2009
		Anencephaly	1.2	[0.7; 2.0]	
	CC, 1999–2003, USA	Overall NTDs	0.9	[0.6; 1.4]	Grewal et al. 2009
	CC, 1989–1991, USA		**1.5**	**[1.0; 2.2]**	**Wasserman et al. 1998**
Operator/laborer	CC, 1999–2003, USA	Spina bifida	1.3	[0.8; 2.2]	Grewal et al. 2009
		Anencephaly	1.1	[0.7; 2.0]	
	CC, 1999–2003, USA	Overall NTDs	1.3	[0.8; 1.9]	Grewal et al. 2009
	CC, 1989–1991, USA		**1.8**	**[1.2; 2.7]**	**Wasserman et al. 1998**
Crowding	CC, 1999–2003, USA	Spina bifida	1.3	[0.8; 2.1]	Grewal et al. 2009
		Anencephaly	0.9	[0.5; 1.7]	
	CC, 1999–2003, USA	Overall NTDs	1.1	[0.7; 1.7]	Grewal et al. 2009
	CC, 1989–1991, USA		**1.7**	**[1.2; 2.6]**	**Wasserman et al. 1998**
Rental occupancy	CC, 1999–2003, USA	Spina bifida	0.8	[0.5; 1.2]	Grewal et al. 2009
		Anencephaly	0.8	[0.5; 1.5]	
	CC, 1999–2003, USA	Overall NTDs	0.8	[0.5; 1.2]	Grewal et al. 2009
	CC, 1989–1991, USA		**1.3**	**[1.0; 1.8]**	**Wasserman et al. 1998**
Specific socioeconomic index	CC, 1999–2003, USA	Spina bifida	1.7	[0.7; 4.4]	Grewal et al. 2009
		Anencephaly	0.6	[0.1; 2.9]	
	CC, 1999–2003, USA	Overall NTDs	1.3	[0.5; 3.0]	Grewal et al. 2009
	CC, 1989–1991, USA		**3.2**	**[1.9; 5.4]**	**Wasserman et al. 1998**
Carstairs Index	CC, 1986–1993, UK	Overall NTDs	1.23	[0.63; 2.37]	Vrijheid et al. 2000
UBN Index	CC, 1992–2001, Argentina	Spina bifida	0.76	[0.52; 1.10]	Pawluk et al. 2014
		Anencephaly	0.93	[0.65; 1.34]	

#### Meta-analysis for NTDs

**Fig 3:**
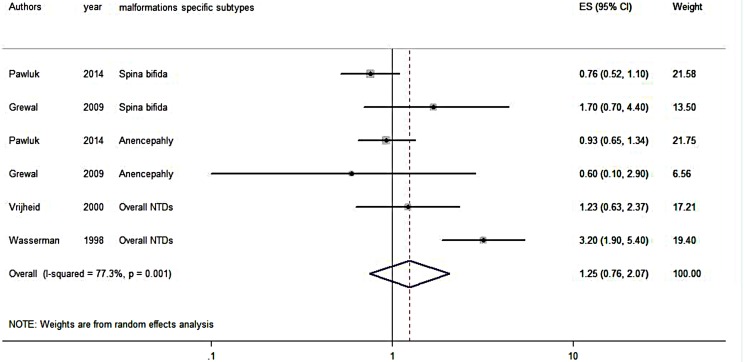
Forest plot on the effect of neighborhood deprivation NTDs. Caption ES: Effect Size; CI: Confidence Interval

Altogether, six results regarding deprivation and NTDs were included in the meta-analysis, showing heterogeneity (I^2^ = 77.3%; p = 0.001). Using random-effect models, OR was not significant among women living in a deprived neighborhood (OR = 1.25, 95% CI: 0.76, 2.07) (Fig 3).

#### Systematic review findings for OFCs

Three studies reported results on OFCs overall; Vrijheid *et al*. in 2000 found a risk close to unity, (OR = 0.95, 95% CI: 0.44, 2.05) [28] whereas risk was statistically significant in Durning *et al*. in 2007 (OR = 1.55, 95% CI: 1.18, 2.04)[25] and in Clark *et al*. in 2013 (OR 2.33, 95% CI: 1.45, 3.74) [26] (Table 6).

**Table 6:** Risk associated with OFCs for different neighborhood socioeconomic indicators.

Neighborhood deprivation	Design, date, location	Malformation(s)	OR	95% CI	Authors, Year
Education	CC, 1999–2008, Texas	Cleft lip with/without cleft palate	**1.19**	**[1.04; 1.36]**	**Lupo et al. 2015**
	CC, 1999–2004, USA		1.1	[0.8; 1.6]	Carmichael et al. 2009
	CC, 1987–1989, USA		1.2	[0.7; 1.9]	Carmichael et al. 2003
	CC, 1999–2008, Texas	Cleft palate	0.90	[0.74; 1.09]	Lupo et al. 2015
	CC, 1999–2004, USA		0.6	[0.4; 1.0]	Carmichael et al. 2009
	CC, 1987–1989, USA		1.4	[0.7; 2.7]	Carmichael et al. 2003
Employment	CC, 1999–2008, Texas	Cleft lip with/without cleft palate	**1.16**	**[1.02;1.31]**	**Lupo et al. 2015**
	CC, 1999–2004, USA		1.0	[0.7; 1.4]	Carmichael et al. 2009
	CC, 1987–1989, USA		0.7	[0.5; 1.1]	Carmichael et al. 2003
	CC, 1999–2008, Texas	Cleft palate	0.87	[0.72;1.05]	Lupo et al. 2015
	CC, 1999–2004, USA		**0.5**	**[0.3; 0.8]**	**Carmichael et al. 2009**
	CC, 1987–1989, USA		0.9	[0.5; 1.7]	Carmichael et al. 2003
Poverty	CC, 1999–2008, Texas	Cleft lip with/without cleft palate	**1.16**	**[1.02;1.32]**	**Lupo et al. 2015**
	CC, 1999–2004, USA		0.9	[0.6; 1.3]	Carmichael et al. 2009
	CC, 1987–1989, USA		0.8	[0.5; 1.3]	Carmichael et al. 2003
	CC, 1999–2008, Texas	Cleft palate	0.87	[0.72;1.06]	Lupo et al. 2015
	CC, 1999–2004, USA		0.6	[0.4; 1.0]	Carmichael et al. 2009
	CC, 1987–1989, USA		1.0	[0.5; 1.9]	Carmichael et al. 2003
Operator/laborer	CC, 1999–2008, Texas	Cleft lip with/without cleft palate	**1.23**	**[1.08;1.40]**	**Lupo et al. 2015**
	CC, 1999–2004, USA		1.1	[0.8; 1.6]	Carmichael et al. 2009
	CC, 1987–1989, USA		1.2	[0.8; 1.9]	Carmichael et al. 2003
	CC, 1999–2008, Texas	Cleft palate	0.96	[0.80;1.16]	Lupo et al. 2015
	CC, 1999–2004, USA		0.7	[0.5; 1.1]	Carmichael et al. 2009
	CC, 1987–1989, USA		1.7	[0.9; 3.0]	Carmichael et al. 2003
Crowding	CC, 1999–2008, Texas	Cleft lip with/without cleft palate	1.04	[0.91;1.20]	Lupo et al. 2015
	CC, 1999–2004, USA		1.2	[0.8; 1.8]	Carmichael et al. 2009
	CC, 1987–1989, USA		1.2	[0.7; 1.9]	Carmichael et al. 2003
	CC, 1999–2008, Texas	Cleft palate	0.99	[0.81;1.21]	Lupo et al. 2015
	CC, 1999–2004, USA		0.7	[0.4; 1.1]	Carmichael et al. 2009
	CC, 1987–1989, USA		0.8	[0.4; 1.5]	Carmichael et al. 2003
Rental occupancy	CC, 1999–2008, Texas	Cleft lip with/without cleft palate	**1.14**	**[1.01;1.28]**	**Lupo et al. 2015**
	CC, 1999–2004, USA		1.1	[0.8; 1.5]	Carmichael et al. 2009
	CC, 1987–1989, USA		1.0	[0.6; 1.6]	Carmichael et al. 2003
	CC, 1999–2008, Texas	Cleft palate	1.07	[0.90;1.28]	Lupo et al. 2015
	CC, 1999–2004, USA		1.0	[0.6; 1.6]	Carmichael et al. 2009
	CC, 1987–1989, USA		1.0	[0.5; 1.9]	Carmichael et al. 2003
Specific socioeconomic index	CC, 1999–2008, Texas	Cleft lip with/without cleft palate	**1.2**	**[1.05;1.37]**	**Lupo et al. 2015**
	CC, 1999–2004, USA		0.9	[0.6; 1.3]	Carmichael et al. 2009
	CC, 1987–1989, USA		0.8	[0.4; 2.0]	Carmichael et al. 2003
	CC, 1999–2008, Texas	Cleft palate	0.94	[0.78;1.14]	Lupo et al. 2015
	CC, 1999–2004, USA		0.8	[0.5; 1.3]	Carmichael et al. 2009
	CC, 1987–1989, USA		0.8	[0.2; 2.8]	Carmichael et al. 2003
Carstairs Index	Co, 1982–2003, Wales	Overall OFCs	**1.55 2.33**	**[1.18; 2.04] [1.45; 3.74]**	**Durning et al. 2007; Clark et al. 2003**
	CC, 1986–1993, UK		0.95	[0.44; 2.05]	Vrijheid et al. 2000
	CC, 1989–1998, Scotland	Cleft lip with/without cleft palate	1.44 **2.33**	[0.98; 2.11]; **[1.23; 4.43]**	Durning et al. 2007; **Clark et al. 2003**
	CC, 1986–1993, UK		0.97	[0.36; 2.63]	Vrijheid et al. 2000
	CC, 1989–1998, Scotland	Cleft palate	1.48 **2.33**	[0.99; 2.22]; **[1.16; 4.71]**	Durning et al. 2007; **Clark et al. 2003**
	CC, 1986–1993, UK		0.95	[0.29; 3.09]	Vrijheid et al. 2000
UBN Index	CC, 1992–2001, Argentina	Cleft lip with/without cleft palate	**1.32**	**[1.01; 1.73]**	**Pawluk et al. 2014**
	CC, 1992–2001, Argentina	Cleft palate	1.69	[0.96; 2.96]	Pawluk et al. 2014

The results of those studies focusing on the specific subtypes cleft lip with/without CLP were contrasting between studies. Several results showed a significantly increased risk of CLP when women live in a deprived region, (OR = 1.32, 95% CI: 1.01, 1.73) [23] and neighborhood (OR = 2.33, 95% CI: 1.23, 4.43) [26]. Lupo *et al*. revealed in 2015 [22] that risk of CLP increased significantly with the level of neighborhood socioeconomic deprivation, using a variety of socioeconomic indicators: deprivation index (OR = 1.20, 95% CI: 1.05, 1.37), rental occupancy (OR = 1.14, 95% CI: 1.01, 1.28), service or production occupation (OR = 1.23, 95% CI: 1.08, 1.40), unemployment (OR = 1.16, 95% CI: 1.02, 1.31), no high school diploma (OR = 1.19, 95% CI: 1.04, 1.36) and poverty (OR = 1.16, 95% CI: 1.02, 1.32). Other results did not report any significant associations[9,27].

Contrasting results were also found among studies focusing on CP phenotype subtypes. Most findings did not report significant associations although some results showed an increased risk of CP among women living in a deprived neighborhood (OR = 2.33, 95% CI: 1.16, 4.71) [26] while others showed inverse significant associations with low employment (OR = 0.5, 95% CI: 0.3, 0.8), education level (OR = 0.6, 95% CI: 0.4, 1.0) or poverty (OR = 0.6, 95% CI: 0.4, 1.0)[9]. In their recent study (published in 2015), Lupo *et al*. found no significant association between neighborhood socioeconomic indicators and risk of CP [22].

#### Meta-analysis for OFCs

**Fig 4:**
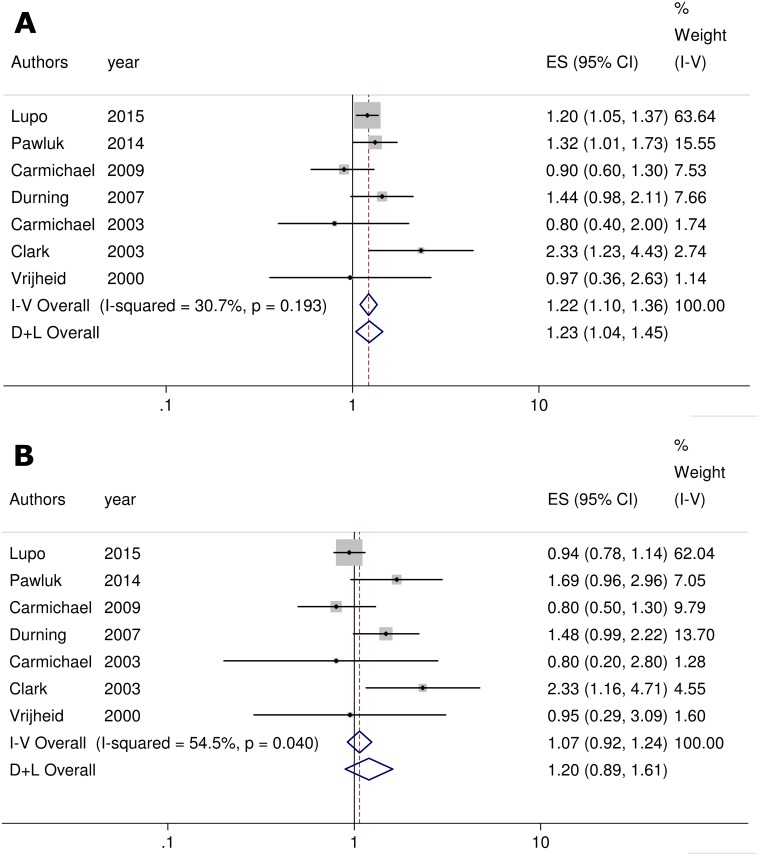
**A) Forest plot on the effect of neighborhood deprivation on CLP.** Caption ES: Effect Size; CI: Confidence Interval **B) Forest plot on the effect of neighborhood deprivation on CP.** Caption ES: Effect Size; CI: Confidence Interval

7 results regarding deprivation and CLP were included in the meta-analyses (Fig 4a), as well as seven results regarding CP (Fig 4b). There was no significant heterogeneity in the summary-effect estimates for CLP (I^2^ = 30.7%; p = 0.193), whereas it was significant for CP (I^2^ = 54.5%; p = 0.04). These values recommend the used of the fixed and random-effect model, respectively.

The summary estimate is significant, with an increased risk of CLP in relation to living in a deprived area (OR = 1.22, 95% CI: 1.10, 1.36) (Fig 4a). No significant result was obtained for CP (OR = 1.20, 95% CI: 0.89, 1.61) (Fig 4b).

## Discussion

### Main findings

This study examined the impact of the SES of pregnant women, measured at a neighborhood or regional level, on congenital abnormalities. The systematic review, conducted on 11 (out of a total of 245) articles suggests an increased risk with the neighborhood deprivation level for both the CHD and NTD malformation groups (not confirmed by the meta-analysis), whereas results were more mixed for OFCs. Our meta-analyses, conducted on a limited number of nine of the 11 articles initially selected included 30 results regarding deprivation and malformations (10 groups and subtypes for CHD, six for NTD, seven for CLP and seven for CP). We confirm previous findings concerning elevated risk for CHD (OR = 1.10, 95% CI: 0.81, 1.5) or NTD (OR = 1.25, 95% CI: 0.76, 2.07). Regarding CLP, we obtain a significantly higher risk in deprived neighborhoods (OR = 1.22, 95% CI: 1.10, 1.36) whereas the risk is not significant for CP (OR = 1.20, 95% CI: 0.89, 1.61). Taking into account the characteristics of the different studies (design, adjustment, definition of the outcomes ….) did not change the meta-risks estimated with the classical meta-analysis approach (S1 text, S1, S2, S3 and S4 Tables).

To our knowledge, this is the first meta-analysis to investigate an association between neighborhood deprivation and CHDs, NTDs and orofacial clefts, based on about 10,000 cases.

These findings are in line with previous results from a meta-analysis performed on individual socioeconomic variables which reported that maternal educational attainment (RR = 1.11, 95%CI: 1.03, 1.21), household income (RR = 1.05, 95%CI: 1.01, 1.09) and maternal occupation (RR = 1.51, 95%CI: 1.02, 2.24) were associated with an increased risk of CHDs[7]. Other studies investigating maternal characteristics have reported that socioeconomic level has been associated with higher risk of a newborn with an NTD [10]. Studies of CLP and CP tend to show no significant association[26,27,31].

Numerous studies investigating relations between other perinatal outcomes such as low birth weight, infant and neonatal mortality have reported increased risk in line with level of deprivation[16,18,32–34]. Some studies have illustrated the mechanisms through which contextual socioeconomic deprivation might influence the risk of infant mortality: socioeconomic level could be related to regularity of medical consultation as well as to compliance with preventive and major nutritional-hygienic practices[32]. Our findings concerning congenital anomalies could be explained through similar conditions acting on early fetal growth; further investigations are needed to support this hypothesis.

Interpretation of our findings must consider weaknesses that could affect the strength of the associations, yield limitations in comparisons or impede the formulation of accurate conclusions. These weaknesses, discussed below, are inherent to (i) outcome data, including the definition and geocoding of data, (ii) neighborhood assessment, and (iii) assessment of the relationship between neighborhood deprivation and malformations. In addition, beyond these factors, the meta-analysis we conducted also faced several methodological limitations.

### Outcome data features

Outcome data may be a source of bias in several ways that could influence both numerator and denominator of the malformations prevalence estimation, resulting in difficulty in achieving accurate comparisons or drawing firm conclusions [35].

First, population study definitions differed between studies, having a possible impact on association measures. Some authors studied all live births and fetal deaths (including pregnancy terminations), whereas others had information only on live births[13,25,26,29], thus restricting ascertainment of birth defects. Not including malformations resulting in early fetal death or elected termination may yield inaccuracy in risk estimates. This tends to bias the direction of the true association because the rate of some anomalies, such as anencephaly, is higher during the fetal period of fewer than 28 weeks of gestational age [35], so that the risk of malformation tends to be higher among stillbirths than among live births[36].

In the same order, inclusion or exclusion of pregnancies terminated prior to 20 weeks of gestational age or without vital records influences estimated malformation prevalence and might, taken to the extreme, reverse the direction of the true association. This might occur when women are less likely to terminate pregnancies as a result of less frequent usage of prenatal diagnosis, lack of access to safe delivery facilities (e.g., poor women)[37], or cultural or religious practices[38–40].

Another source of limitation lies in the databases. Registries could be more exhaustive than hospital records. Specific hospitals generally have no distinct catchment areas and, hence, no defined denominator of the population from which all cases are ascertained. In addition, home births may not be reported and subsequently be undercounted, occasioning referral bias[35].

Classification of malformations is heterogeneous between studies, making comparisons difficult. In this review, while certain authors use a universal standard International Classification of Disease (version 9 and 10) [11,13,28,29], others use local ones[9,27]. The fact that other studies have not reported the classification they used precludes proper identification of misclassifications. Similarly, specific definition of defects may ease detection of causal associations. Broad groupings of malformations into all congenital anomalies combined such as CHDs and NTDs, may also have hampered the ability to analyze associations for specific malformation phenotypes by diluting relevant cases. One such example is the grouping of CHDs: some authors assessed CHDs as a whole [28,29] whereas in other studies, subtypes such as TOF and dTGA [9,27] or VSD and truncus arteriosus [23] were assessed as independent variables.

Irrespective of the limitations below, the process of data geocoding may also introduce some uncertainty. In several studies, a high number of addresses were either unreported, or out of the country or study area. Omission of non-geocoded cases could distort associations in cohort study designs—should this be differential according to deprivation characteristics. With regard to case-control studies, this could be a limitation only because non-geocoded cases are disproportionately lower for socioeconomic determinants (below high school education level, and lower income) than non-geocoded controls, or conversely [24].

### Neighborhood assessment

When studies explore the neighborhood effect, a particular source of uncertainty is due to the borders of the study areas—an issue that includes scale and aggregation effects. These challenges are known as Modifiable Area Unit Problems (MAUP)[41,42]. These MAUPs will bring about changes in the apparent geographical distribution of the variable in question, thus influencing the magnitude of effects observed. These two phenomena are often taken into account in geographical studies[43], though rarely in spatial epidemiology. The MAUP affects descriptive statistics, including variances and coefficients of correlation and regression[44]. For instance, among the studies included in this review, the scale ranges from region[23] to neighborhood [9,13,27–29]. Definition of neighborhood itself also affects comparisons, since it varies across studies from about 5,000 to 10,000 households[9] to an average of 282 households[13,29].

One other source of limitation lies in how the neighborhood is characterized. The variety of contextual indicators is a major concern. Some authors used only single variables (income, poverty, education, etc.) while others (Carstairs, Townsend and others) accommodated composite indices. Besides, comparisons between composite socioeconomic indices are also complex and depend on the make-up of the census variables. Indices may have been underpowered for the detection of any SES influence, given that the relative role played by each single neighborhood variable (income, unemployment, and education, for instance) is different and can mask real associations.

### Assessment of the relation between neighborhood deprivation and malformations

An array of factors will be evoked below. Firstly, the various confounding factors included in the individual studies could influence both effect sizes and comparisons between studies. Some studies did not use any covariates [26], while others have used up to four putative confounders, including baby and maternal characteristics and healthy behaviors among others [9,11,22,27]. An absence of systematic adjustment for commonly known factors may also impair comparisons—for instance folic acid supplementation, which is known to decrease the risk of NTDs[45–47], but OFCs[48] and CHDs[49] tool.

Differences in statistical methods used may obscure interpretation of the results as a whole. Some authors used conventional logistic regression instead of multilevel regression—viewed as a useful tool and more appropriate to the investigation of contextual effect[50]. Indeed, a conventional approach allocates the contextual effect to the individual level, whereas multilevel analysis strives to distinguish both the characteristics of individuals and those of the place where they live. This approach is now acknowledged to be more appropriate to assessment of the true contextual effect related to health[15].

Various features of the studies—such as study population, the classification and definition of malformations, neighborhood assessment and confounding factors—could impact the quality of each study included in the meta-analysis, undermining the value of the combined estimates.

The meta-analysis approach has several methodological flaws. The first of these deals with sample size. Performing these analyses across eight studies or fewer is a limitation. This was deemed acceptable by Davey *et al*., in 2011, who reviewed 22,453 meta-analyses from the Cochrane Database of Systematic Reviews and reported that about 75% of the meta-analyses in each of the categories (“gynecology, pregnancy and birth”, “obstetrics outcomes” and “continuous data type”) contained no more than five studies[51]. The vast majority of the studies included in this meta-analysis were of case-control design, which is subject to recall and selection bias. Also, our work may be impacted by the publication bias that is inherent to the systematic literature review procedure. According to the 10.4.3.1 recommendations from the Cochrane Handbook for Systematic Reviews of Interventions, Version 5.1.0, at least ten studies are required in order to draw an interpretable funnel plot[21]. Unfortunately, the number of studies included in our meta-analyses did not reach that number. However, we do not think our results were biased by the inclusion of the same study several times over, because the meta-analysis was stratified into four groups by malformation type—a strategy already used by other authors[7].

## Conclusion

Our systematic review and meta-analysis provide evidence of an association between low neighborhood SES level and an increased risk of CLP phenotypes. Any general conclusions must be considered carefully, given that deprivation is a broad indicator that could reflect a variety of exposure factors (low SES might be associated with exposure to harmful environmental contaminants, lifestyle factors, lack of antenatal prevention, limited access to health services etc.). Hence, the associations this study suggests should trigger the generation of hypotheses for the design of new studies. This study does not offer precise knowledge about biologic underpinning. In clinical planning, however, this study highlighted the usefulness of taking into account the area in which pregnant women live in order to promote preventive messages in medical care during pregnancy.

Furthermore, in order to reveal contextual effect, we encourage further investigations taking account of both individual and neighborhood characteristics, using accurate statistical modeling—such as multilevel analysis approaches.

## Supporting Information

S1 Table Characteristics of the included studies regarding CHDs: the scores for each criterion and the quality index. (DOCX)

S2 Table Characteristics of the included studies regarding NTDs: the scores for each criterion and the quality index. (DOCX)

S3 Table Characteristics of the included studies regarding OFCs: the scores for each criterion and the quality index. (DOCX)

S4 Table Combined effect of neighborhood deprivation on congenital heart defects (CHDs), on neural tube defects (NTDs) and orofacial clefts (OFCs) taking into account the Quality index (Qi). (DOCX)

S1 Text Quality effect model methods. (DOCX)

## Abbreviations

ASD: Atrial Septal Defect
CHD: Congenital Heart Defects
CLP: Cleft lip with/without Cleft Palate
CP: Cleft Palate
ES: Effect Size
MAUP: Modifiable Area Unit Problems
NTD: Neural Tube Defects
OR: Odds Ratios
RR: Relative Risks
SB: Spina Bifida
SES: Socioeconomic status
TOF: Tetralogy Of Fallot
VSD: Ventricular Septal Defect
OFC: OroFacial Clefts
PRISMA: Preferred Reporting Items for Systematic reviews and Meta-Analyses
