# Supplementary material for: Neighborhood Deprivation and Risk of Congenital Heart Defects, Neural Tube Defects and Orofacial Clefts: A Systematic Review and Meta-Analysis
Source: PLoS One. 2016 Oct 26;11(10):e0159039. doi: 10.1371/journal.pone.0159039 (PMC5082651; doi:10.1371/journal.pone.0159039)
Supplement: S1 Table — (DOCX) [file pone.0159039.s001.docx]

**S1 Table. Characteristics of the included studies regarding CHDs : the scores for each criterion and the quality index**

|  | Pawluk MS 2014 | Carmichael SL 2009 | Carmichael SL 2003 | Vrijheid M 2000 |
| --- | --- | --- | --- | --- |
| Sample size | 13,836 (1) | 894 (1) | 524 (1) | 230 (1) |
| Design | CC (0.75) | CC (0.75) | CC (0.75) | CC (0.75) |
| Country | Argentina (1) | USA (1) | USA (1) | United kingdom (1) |
| Timeframe | 1992-2001 (1) | 1999 -2004 (1) | 1987-89 (1) | 1986-93 (1) |
| Geocodage rate | Not reported (0.75) | 90% for cases and 88% for controls (1) | More than 97% for cases and controls (1) | More than 99.9% for cases and controls (1) |
| Definition of CA | NC (0.5) | Coding based on ICD9 (0.75) | Coding based on Clark classification (0.75) | ICD 9 ; ICD 10 (1) |
| Assessment of CA | Register (1) | Hospital reports and medical records (0.75) | Register (1) | Registers (1) |
| Assessment of SE | socioeconomic index based on UBN value (1) | Validated socioeconomic index (1) | Validated socioeconomic index (1) | Carstair deprivation index (1) |
| Adjustments for covariates | - Mothers characteristics: age, gravidity order, native descent  - Mothers behavior: number of antenatal visits  (0.75) | - Mothers characteristics:  race-ethnicity , body mass index  - Mothers behavior: intake of folic acid-containing supplements, smoking, binge drinking (0.75) | - Mothers characteristics:  Race-ethnicity  - Mothers behavior  Vitamin use, smoking, binge drinking (0.75) | - Neighborhood characteristics: distance of residence from a landfill  - Mother’s characteristics: age  (0.5) |
| Effect size | OR – no transformation (1) | OR – no transformation (1) | OR – no transformation (1) | OR – no transformation (1) |
| Quality index | 0.875 | 0.9 | 0.925 | 0.925 |
