# Supplementary material for: Neighborhood Deprivation and Risk of Congenital Heart Defects, Neural Tube Defects and Orofacial Clefts: A Systematic Review and Meta-Analysis
Source: PLoS One. 2016 Oct 26;11(10):e0159039. doi: 10.1371/journal.pone.0159039 (PMC5082651; doi:10.1371/journal.pone.0159039)
Supplement: S2 Table — (DOCX) [file pone.0159039.s002.docx]

**S2 Table. Characteristics of the included studies regarding NTDs : the scores for each criterion and the quality index**

|  | Pawluk MS 2014 | Grewal 2009 | Vrijheid M 2000 | Wasserman 1998 |
| --- | --- | --- | --- | --- |
| Sample size | 13,974 (1) | 835 (1) | 107 (0.75) | 977 (1) |
| Design | CC (0.75) | CC (0.75) | CC (0.75) | CC (0.75) |
| Country | Argentina (1) | USA (1) | United kingdom (1) | USA (1) |
| Timeframe | 1992-2001 (1) | 1999-2003 (1) | 1986-93 (1) | 1989-1991 |
| Geocodage rate | Not reported (0.75) | 84 % of eligible case mothers / 88% of control mothers (1) | More than 99.9% for cases and controls (1) | 89% for cases and 92% for controls (1) |
| Definition of CA | NC (0.5) | NC (0.5) | ICD 9 ; ICD 10 (1) | ICD 9 (1) |
| Assessment of CA | Register (1) | Multiple hospital reports and medical records (0.75) | Registers (1) | Registers (1) |
| Assessment of SE | socioeconomic index based on UBN value (1) | Validated socioeconomic index (1) | Carstair deprivation index (1) | Based on a new socioeconomic index (0.75) |
| Adjustments for personal covariates | - Mothers characteristics: age, gravidity order, native descent  - Mothers behavior: number of antenatal visits (0.75) | - Mothers characteristics:  Age, body mass index, gravidity, race-ethnicity  - Mothers behavior  Intake of folic acid-containing supplements (0.75) | - Neighborhood characteristics: distance of residence from a landfill  - Mother’s characteristics: age  (0.5) | - Mother’s characteristics:  race/ethnicity, age, BMI, fever, education, household income, employment, family occupation  - Mothers behavior: periconceptional vitamin use (0.75) |
| Effect size | OR – no transformation (1) | OR – no transformation (1) | OR – no transformation (1) | OR – no transformation (1) |
| Quality index (Qi) | 0.875 | 0.875 | 0.9 | 0.825 |
