# Supplementary material for: Neighborhood Deprivation and Risk of Congenital Heart Defects, Neural Tube Defects and Orofacial Clefts: A Systematic Review and Meta-Analysis
Source: PLoS One. 2016 Oct 26;11(10):e0159039. doi: 10.1371/journal.pone.0159039 (PMC5082651; doi:10.1371/journal.pone.0159039)
Supplement: S3 Table — (DOCX) [file pone.0159039.s003.docx]

**S3 Table. Characteristics of the included studies regarding OFCs : the scores for each criterion and the quality index**

|  | Lupo PJ, 2015 | Pawluk MS 2014 | Carmichael SL 2009 | Durning P, 2007 | Carmichael SL 2003 | Clark JD 2003 | Vrijheid M 2000 |
| --- | --- | --- | --- | --- | --- | --- | --- |
| Sample size | 18,402 (1) | 13,962(1) | 1,225 (1) | 831 (1) | 993 (1) | 834 (1) | 73 (0.75) |
| Design | CC(0.75) | CC (0.75) | CC (0.75) | Eco (0.5) | CC (0.75) | Eco (0.5) | CC (0.75) |
| Country | USA (1) | Argentina (1) | USA (1) | Wales (1) | USA (1) | Scotland (1) | United kingdom (1) |
| Timeframe | 1999-2008 (1) | 1992-2001 (1) | 1999 -2004 (1) | 1982-2003 (1) | 1987-89 (1) | 1989 to 1998 (1) | 1986-93 (1) |
| Geocodage rate | 93% for cases and controls (1) | Not reported (0.75) | 90% for cases and 88% for controls (1) | 97.8% for cases and 99.97% for denominator (1) | More than 97% for cases and controls (1) | Not reported (0.75) | More than 99.9% for cases and controls (1) |
| Definition of CA | National Birth Defects prevention  Study (0.75) | NC (0.5) | Coding based on ICD9 (0.75) | NC (0.5) | Coding based on Clark classification(0.75) | NC (0.5) | ICD 9 ; ICD 10 (1) |
| Assessment of CA | Register (1) | Register (1) | Hospital reports and medical records (0.75) | Register (1) | Register (1) | Register (CLEFTSiS) (1) | Registers (1) |
| Assessment of SE | Validated socioeconomic index (1) | socioeconomic index based on UBN value (1) | Validated socioeconomic index (1) | Townsend index (1) | Validated socioeconomic index (1) | Carstair deprivation index (1) | Carstair deprivation index (1) |
| Adjustments for personal covariates | Babies characteristics: sex and birth of year  - Mothers characteristics: age, race/ethnicity, education,  - Mothers behavior: smoking (1) | - Mothers characteristics: age, gravidity order, native descent  - Mothers behavior: number of antenatal visits (0.75) | - Mothers characteristics:  race-ethnicity , body mass index  - Mothers behavior: intake of folic acid-containing supplements, smoking, binge drinking (0.75) | None (0) | - Mothers characteristics:  Race-ethnicity  - Mothers behavior  Vitamin use, smoking, binge drinking (0.75) | None (0) | - Neighborhood characteristics: distance of residence from a landfill  - Mother’s characteristics: age  (0.5) |
| Effect size | OR – no transformation (1) | OR – no transformation (1) | OR – no transformation (1) | RR🡪OR (0.75) | OR – no transformation (1) | RR🡪OR (0.75) | OR – no transformation (1) |
| Quality index (Qi) | 0.95 | 0.975 | 0.9 | 0.775 | 0.925 | 0.75 | 0.9 |
