# Supplementary material for: Neighborhood Deprivation and Risk of Congenital Heart Defects, Neural Tube Defects and Orofacial Clefts: A Systematic Review and Meta-Analysis
Source: PLoS One. 2016 Oct 26;11(10):e0159039. doi: 10.1371/journal.pone.0159039 (PMC5082651; doi:10.1371/journal.pone.0159039)
Supplement: S4 Table — (DOCX) [file pone.0159039.s004.docx]

**S4 table . Combined effect of neighborhood deprivation on congenital heart defects (CHDs), on neural tube defects (NTDs) and orofacial clefts (OFCs) taking into account the Quality index (Qi).**

| **Congenital malformations** | **Combined effect** | **95% Confidence Interval** |
| --- | --- | --- |
| CHDs | OR=1.04 | [0.74 ; 1.44] |
| NTDs | OR=1.12 | [0.64 ; 1.95] |
| OFCs CLP | OR=1.21 | [1.01 ; 1.46] |
| CP | OR=1.18 | [0.72 ; 1.94] |

|  |  |  |  |
| --- | --- | --- | --- |
|  |  |  |  |
