# Supplementary material for: Neighborhood Deprivation and Risk of Congenital Heart Defects, Neural Tube Defects and Orofacial Clefts: A Systematic Review and Meta-Analysis
Source: PLoS One. 2016 Oct 26;11(10):e0159039. doi: 10.1371/journal.pone.0159039 (PMC5082651; doi:10.1371/journal.pone.0159039)
Supplement: S1 Text — (DOCX) [file pone.0159039.s005.docx]

**S1 Text. Quality effect model methods**

Individual quality assessment methodology was adapted from Croteau *et al* in 2009 and doi and Thalib in 2008. The checklist was defined by researcher consensus. It assigned a maximum of 1.00 point for the different methodological criteria and a quality score (Qi=$( \frac{\sum_{1}^{10} score criteria}{10}$)) is calculated for each study included in the meta-analysis.

Ten Criteria are defined as follow:

- **Sample size**

(**1**): completely satisfactory/ justified by power analysis; (**0.5**): somewhat satisfactory; (**0**): not sufficient/not justified.

- **Design**

(**1**): cohort; (**0.75**): case-control; (**0.5**): ecological

- **Country** where the study was carried out

(**1**): With good working and living conditions/high socio-economic standard; (**0.5**): Difficult conditions/lower socio-economic standard; (**0.25**): Very difficult conditions/very low socio-economic standard; (**0**): Not reported.

(1: USA, UK, Sweden, Latina America; 0.5: China)

- **Timeframe**

(**1**): Reported; (**0**): Not reported.

- **Geocodage rate**

(**1**): ≥80%/considerable part of the population; (**0.75**): Not reported.

- **Definition of congenital anomalies**

(**1**): ICD; (**0.75**): Others classifications; (**0.5**): No detailed.

- **Assessment of congenital anomalies**

(**1**): registers; (**0.75**): medical records/physician’s exam/valid database; (**0.5**): self-report; (**0**): not specified.

- **Assessment of the socioeconomic index**:

(**1**): official/validated index; (**0.75**): study-specific index.

- **Adjustments for covariates** (cov) *(table 1)*

(**1**): At least 1 (cov) in each of the three covariates groups (baby’s characteristic, mother’s characteristics, mother’s behavior); (**0.75**): At least 1 (cov) baby’s characteristic and at least 1 (cov) mother’s characteristic (or mother’s behavior); (**0.5**): At least 1 (cov) in one of the three covariate groups (baby’s characteristic, mother’s characteristic, mother’s behavior); (**0**): no covariates

- **Effect size calculation for meta-analysis based on odds ratios**

(**1**): no transformations and no data imputation; (**0.75**): mild transformation and no data imputation; (**0.5**): several transformations and no data imputation; (**0.25**): considerable transformations and data imputation
